# Evaluation of Liver Biomarker Levels in Farmers Occupationally Exposed to Pesticides: A Cross-Sectional Study

**DOI:** 10.3390/diseases14090337

**Published:** 2026-09-14

**Authors:** Hung The Dang, Thoa Phuong Nguyen, Thu Thi Yen Pham, Chinh Thi Luu, Ha Thi Thu Nguyen, Oanh Thi Kieu Nguyen, Oanh Thi Phuong Ngo, Chinh Thi Tuyet Do, Ha Thi Ngoc Bui, Quan Hong Duong

**Affiliations:** Laboratory Center, Hanoi University of Public Health, Hanoi 100000, Vietnam

**Keywords:** pesticide exposure, liver biomarkers, occupational health, fruit farmers, hepatotoxicity

## Abstract

Background/Objectives: Occupational pesticide exposure is a recognized hepatotoxic hazard; however, biomonitoring data characterizing liver function among Vietnamese agricultural workers remain scarce. This study compared serum hepatic biomarkers between pesticide-exposed fruit farmers (exposed group) and a non-exposed reference group (unexposed group) in a northern province in Vietnam, and evaluated whether biomarker alteration varied with cumulative exposure duration. Methods: A cross-sectional study using convenience sampling was conducted from April to October 2022 among 150 adults, comprising 100 fruit farmers with ≥1 year of direct occupational pesticide exposure and 50 unexposed controls. Serum aspartate aminotransferase (AST), alanine aminotransferase (ALT), total and indirect bilirubin, alpha-fetoprotein (AFP), and glucose were quantified using clinical laboratory assays. Multivariable linear regression models, adjusting for age, sex, and body mass index (BMI), were used to evaluate associations between pesticide exposure duration and biomarker levels. Results: Pesticide-exposed farmers had significantly higher mean concentrations of ALT (28.88 vs. 22.36 U/L, *p* = 0.002), AFP (3.06 vs. 1.16 ng/mL, *p* < 0.001), total bilirubin (9.93 vs. 7.74 μmol/L, *p* < 0.001), and indirect bilirubin (8.40 vs. 5.26 μmol/L, *p* < 0.001) than unexposed controls. While mean AST did not differ (*p* = 0.105), the proportion of abnormal AST cases was significantly higher among exposed individuals (23% vs. 8%, *p* = 0.043). After adjustment for age, sex, and BMI in multivariable linear regression models, prolonged exposure (>10 years) remained modestly associated with higher AFP and bilirubin levels (*p* < 0.05), while associations with ALT and AST were not statistically evident (*p* > 0.05). Conclusions: These findings suggest a possible association between occupational pesticide exposure and subclinical hepatic alterations, supporting calls for regular health surveillance and enhanced protective measures among fruit farmers.

## 1. Introduction

Pesticides, comprising insecticides, fungicides, herbicides, and plant growth regulators, are indispensable agrochemical inputs that protect crop yield and quality against pests, pathogens, and weeds. Global pesticide consumption has risen markedly over the past three decades, driven by agricultural intensification to meet the demands of a growing population. An estimated 3.73 million tonnes of pesticides are applied annually worldwide, with a disproportionate share used in low- and middle-income countries [1]. Agriculture remains a vital pillar of Vietnam’s economy; however, the expansion of high-yield commercial fruit production has driven a substantial increase in agrochemical use. Annual pesticide imports range between 70,000 and 100,000 tonnes, incorporating over 500 registered active ingredients across various insecticidal, fungicidal, and herbicidal formulations [2]. Field surveys across agricultural regions in Vietnam frequently document the simultaneous application of multi-component agrochemical mixtures and variable dosing practices, which may heighten chemical exposure complexity alongside challenges related to pesticide quality regulation [3,4].

The hepatotoxic potential of pesticides is well documented at both the mechanistic and epidemiological levels. The liver, as the principal organ of xenobiotic biotransformation, is disproportionately exposed to reactive intermediates generated during hepatic phase I metabolism, predominantly via the cytochrome P450 (CYP) monooxygenase system [5]. In addition to bioactivation-dependent toxicity, accumulating experimental evidence reports that diverse pesticide classes induce hepatocellular oxidative stress, mitochondrial impairment, and regulated cell death mechanisms, such as ferroptosis, necroptosis, and pyroptosis, driving downstream hepatic inflammation, steatosis, and progressive fibrosis [6]. These molecular perturbations are clinically reflected in the elevation of circulating hepatic biomarkers, such as AST, ALT, AFP, and bilirubin fractions, and have been epidemiologically linked to an increased risk of non-alcoholic fatty liver disease and hepatocellular carcinoma among chronically exposed populations [7,8].

Epidemiological studies in various agricultural cohorts report significant elevations in serum AST and ALT levels among pesticide-exposed workers relative to non-exposed comparison groups. Several reports also document secondary alterations in bilirubin fractions, alongside increased risks of clinically hepatobiliary disease [9,10,11,12]. Nevertheless, the extent and specific profile of hepatic alterations vary across studies. Whereas some reports identified no significant differences in transaminase levels between exposed and unexposed groups, others documented isolated increases in ALT without concomitant AST changes. This discrepancy likely stems from differences in exposure duration, application intensity, active chemical ingredients, use of personal protective equipment, and methodological designs [9,10,11,12]. Despite growing global evidence, biomonitoring data evaluating hepatic function in Vietnamese agricultural workers remain limited, as domestic occupational health research has predominantly focused on acute poisoning events and general safety practices rather than subclinical and chronic hepatobiliary alterations [3,4].

This knowledge gap is critical given the burden of liver disease in Vietnam. A recent nationwide study collecting data from nearly all (94.6%) of Vietnam’s commune health stations revealed that liver cancer is the leading cause of cancer mortality, accounting for 27.1% of all cancer deaths (31.04% in men and 19.91% in women) [13]. While this burden is multifactorial, emerging evidence links chronic occupational and environmental chemical toxicities directly to hepatic damage. Recent research shows that even low-level, persistent exposures to environmental toxicants can significantly impair liver health and accelerate disease progression, particularly when acting synergistically with other risk factors [14,15].

Despite the intersection between agricultural pesticide reliance and a national liver disease burden, localized biomonitoring remains unaddressed in key agricultural hubs. The present study was conducted in an established fruit-cultivation region in a northern province of Vietnam, characterized by the intensive production of perennial fruit crops and subtropical orchards. This region represents a setting of plausible high cumulative pesticide exposure; however, no prior study has systematically characterized liver function biomarkers in this high-risk population.

To address this critical gap, the present cross-sectional study was designed to compare serum concentrations of glucose, AST, ALT, total and indirect bilirubin, and AFP between pesticide-exposed fruit farmers and a non-exposed control group. Additionally, we evaluated the association between cumulative exposure duration and the magnitude of hepatic biomarker alteration. We hypothesized that occupational pesticide exposure is associated with a measurable duration-dependent elevation in circulating hepatic biomarkers among pesticide-exposed fruit farmers.

## 2. Materials and Methods

### 2.1. Study Design and Setting

A cross-sectional study was conducted to evaluate liver function biomarkers among pesticide-exposed fruit farmers relative to a non-exposed reference population. The study was carried out from April to October 2022 in an established fruit-cultivation region in a northern province of Vietnam, an area characterized by the intensive commercial production of perennial subtropical fruit orchards and citrus crops. This study is reported in accordance with the STROBE (Strengthening the Reporting of Observational Studies in Epidemiology) statement for cross-sectional studies.

### 2.2. Study Participants and Sample Size

Eligible participants were adults aged over 18 years residing in the study areas. Participants were recruited using convenience sampling and categorized into two groups: an exposed group (n = 100) of fruit farmers actively engaged in cultivation with >1 year of direct pesticide exposure, and a reference group (n = 50) of local residents reporting no agricultural occupation or pesticide contact. The sample size and 2:1 allocation were pragmatic decisions dictated by participant availability during the study period and available research funding. This sample size achieved an estimated statistical power of 81.8%, satisfying the standard 80% threshold.

Exclusion criteria were implemented to control for underlying liver pathology and systemic confounders: (1) self-reported history of liver disease (including viral hepatitis or cirrhosis) or severe chronic diseases; (2) active tobacco smoking or alcohol intake; (3) current pregnancy; and (4) lack of written informed consent. Restricting enrollment to non-smokers and non-drinkers strengthened the internal validity.

### 2.3. Occupational Exposure Characterization

Exposure characterization followed a structured multi-step methodology: (1) direct occupational engagement in pesticide application; (2) an interviewer-administered questionnaire evaluating cumulative years of pesticide handling, seasonal application frequency, and PPE utilization habits; (3) field verification of specific pesticide formulations through direct inspection of container labels and discarded packaging at application sites to reduce recall bias; and (4) toxicological hazard classification of confirmed active ingredients aligned with WHO guidelines [16].

### 2.4. Data Collection Procedures

Trained research staff collected data via structured interviews, capturing socio-demographic variables (age, sex, marital status, residency duration, and education) and detailed occupational pesticide exposure parameters. Height and weight were measured using calibrated devices to calculate BMI (kg/m^2^), which was categorized according to WHO Asian-specific thresholds.

### 2.5. Biomarker Analysis

Blood samples were collected by qualified nurses via venipuncture in the morning before breakfast during the period coinciding with active pesticide spraying and fruit development in the studied areas. A 2 mL blood sample was obtained from each subject using an EDTA vacutainer tube. Following collection, all samples were immediately stored in an ice-cold chamber (2–8 °C) and transported to the laboratory for processing within 6 h of collection. Samples were allowed to clot at room temperature and were subsequently centrifuged to separate serum, which was analyzed using an automatic biochemical and immunoassay analyzer (AU480 Clinical Chemistry and E601 Immunoassay Analyzers, Beckman Coulter, Inc., California, United States) for determination of the following biomarkers, on the basis of standard protocols for clinical laboratories: glucose, AST, ALT, total bilirubin, indirect bilirubin, and AFP. Reference ranges applied for interpretation of each biomarker are presented together with the corresponding results. All blood samples were assigned unique identification codes prior to processing, and laboratory personnel performing the biochemical analyses were fully blinded to the exposure status, demographic data, and group allocation of the participants.

Internal quality control (IQC) procedures followed the laboratory’s documented standard operating procedures (SOPs) to ensure analytical accuracy and consistency. The analyzers were calibrated monthly. Two levels (normal and pathological) of internal quality control (IQC) samples were run alongside the serum samples in each analytical batch. After test analysis, printed results were checked for all post-analytical factors and approved by the responsible laboratory head before release.

### 2.6. Study Variables

Dependent variables comprised continuous serum concentrations and categorical abnormality rates for AST, ALT, total bilirubin, indirect bilirubin, AFP, and glucose. Pesticide exposure status (exposed vs. reference) served as the primary exposure variable. For duration–response analyses, exposure duration was classified into three ordinal categories: <5 years, 5–10 years, and >10 years. Key potential confounders (age, sex, BMI, marital status, and duration of residence) were compared between groups using descriptive statistics to verify cohort comparability.

### 2.7. Statistical Analysis

All data were entered, cleaned, and checked for completeness prior to analysis. Descriptive statistics are presented as mean ± standard deviation (SD) and range (minimum–maximum) for continuous variables, and as frequency (percentage) for categorical variables. The normality of continuous variables was assessed using the Shapiro–Wilk test prior to selecting parametric or non-parametric comparison tests; all biomarker variables analyzed by Student’s t-test met the normality assumption at the group level.

Between-group comparisons (exposed vs. unexposed) for continuous biochemical variables were performed using the independent-samples Student’s *t*-test (Welch’s correction applied where group variances were unequal). Categorical variables, including the proportion of biochemically abnormal values, were compared using the chi-square test or Fisher’s exact test where expected cell counts were below 5, with odds ratios (OR) and 95% confidence intervals (CI) to quantify the strength of association. One-way analysis of variance (ANOVA), with Tukey–Kramer post hoc pairwise comparison, was used to compare biomarker concentrations across the three exposure-duration strata. To control for potential confounders, multivariable linear regression models were performed using serum hepatic biomarkers (ALT, AST, AFP, and bilirubin fractions) as continuous outcome variables. Unstandardized coefficients with 95% confidence intervals (95% CI), and FDR-adjusted p-values were reported. A two-tailed *p*-value < 0.05 was considered statistically significant throughout. All statistical analyses were performed using STATA version 14.0.

### 2.8. Ethical Considerations

The study protocol was reviewed and approved by the Ethics Committee of Hanoi University of Public Health (Decision No. 441/2021/YTCC-HD3, 17 December 2021), in accordance with the ethical principles of the Declaration of Helsinki. Written informed consent was obtained from all participants prior to enrollment, following a full explanation of the study objectives, procedures, potential risks, and the voluntary nature of participation. Participant data were anonymized using unique identification codes, and confidentiality was maintained throughout data collection, laboratory analysis, and reporting.

## 3. Results

### 3.1. Participant Characteristics

Table 1 shows the characteristics of the study population. A total of 150 participants were enrolled, comprising 100 pesticide-exposed fruit farmers and 50 non-exposed reference participants. The two groups were broadly comparable in age (49.4 ± 10.2 vs. 48.9 ± 5.5 years) and BMI (22.8 ± 2.8 vs. 22.7 ± 2.7 kg/m^2^), and all participants in both groups were non-smokers and non-drinkers. The exposed group had a lower proportion of male participants (20.0% vs. 32.0%) and a higher proportion of married individuals (94.0% vs. 90.0%), although neither difference reached statistical significance. All participants in both groups had resided locally for more than 10 years. Age, BMI, sex distribution, and marital status did not differ significantly between groups (all *p* > 0.05).

### 3.2. Exposure and Safety Practice Assessment

The occupational characteristics among the exposed group are summarized in Table 2. High application frequency was prevalent, with 46.0% and 45.0% of farmers applying pesticides >10 times and > 5 times per season, respectively. Regarding personal protection, while 100.0% of the participants reported wearing masks during chemical application, compliance with other individual barrier items varied substantially: protective clothing (47.0%), rubber boots (92.0%), protective gloves (92.0%), hats/head coverings (96.0%), and eye goggles/face shields (11.0%). When evaluating protection across all six essential protective items, only 10.0% of the farmers achieved fully adequate protective practice, leaving 90.0% of the exposed farmers with incomplete barrier protection during pesticide application.

### 3.3. Pesticide Products and Hazard Classification

The pesticide products used by farmers are presented in Table 3. Twenty-seven distinct commercial pesticide products were identified through label verification among the exposed farmers, spanning insecticides (n = 18 products), fungicides (n = 5 products), herbicides (n = 2 products), and plant growth regulators (n = 2 products). The large majority of products were classified as WHO Hazard Class II (“moderately hazardous”) according to the WHO Recommended Classification of Pesticides by Hazard [16], including formulations containing organophosphates (e.g., chlorpyrifos ethyl, quinalphos, profenofos) and pyrethroids (e.g., lambda-cyhalothrin, alpha-cypermethrin, permethrin), while several avermectin-based products were classified as biological-class agents. No Class Ia (“extremely hazardous”) or Class Ib (“highly hazardous”) products were identified among the surveyed farmlands.

### 3.4. Biomarker Test Results

The concentrations of biomarkers for the two groups are presented in Table 4. Serum concentrations of AST, ALT, total and indirect bilirubin, and AFP were consistently higher in the pesticide-exposed group compared with the unexposed group, while glucose level did not differ between groups. Although mean concentrations of all evaluated biomarkers (AST, ALT, total bilirubin, indirect bilirubin, and AFP) fell within normal reference ranges in both study groups, the proportion of abnormal cases was elevated among exposed farmers compared with unexposed controls, with Fisher’s exact tests confirming statistical significance for AST and ALT (*p* = 0.043 and *p* = 0.045, respectively), but not for AFP or bilirubin fractions (*p* < 0.05). The mean AST concentration did not differ significantly between groups (*p* = 0.105); however, the concentrations of ALT, AFP, total bilirubin, and indirect bilirubin were all significantly elevated in the exposed group (*p* = 0.002, *p* < 0.001, *p* < 0.001, and *p* < 0.001, respectively).

### 3.5. Cumulative Exposure Duration and Biomarker Variations

Table 5 showed that mean concentrations of AST, ALT, AFP, total bilirubin, and indirect bilirubin increased across the three exposure-duration strata (<5 years, 5–10 years, >10 years) among pesticide-exposed farmers. One-way ANOVA confirmed a statistically significant exposure-duration effect for AFP, total bilirubin, and indirect bilirubin, whereas the upward trends observed for AST and ALT did not reach conventional statistical significance.

AFP showed a significant exposure-duration gradient (1.06 → 2.06 → 7.08 ng/mL; F = 18.66, *p* < 0.001). Total and indirect bilirubin also increased significantly with exposure duration (*p* = 0.014 and *p* = 0.031, respectively). In contrast, although AST and ALT means increased numerically across strata, substantial within-stratum variability precluded statistical significance (AST: *p* = 0.164; ALT: *p* = 0.139).

As presented in Table 6, the “>10 years” stratum differed significantly from both the “<5 years” and the “5–10 years” strata for AFP and total bilirubin, while the two shorter duration strata did not differ significantly from one another, suggesting that hepatic biomarker elevation becomes apparent only after more than a decade of cumulative occupational pesticide exposure. For indirect bilirubin, only the “>10 years” versus “<5 years” comparison reached significance.

After adjusting for potential confounders in the multivariable linear regression model (Table 7), prolonged exposure (>10 years) remained modestly associated with slight increases in AFP, total bilirubin, and indirect bilirubin (all *p* < 0.05). Conversely, exposure duration exhibited a borderline association with ALT (*p* = 0.060) and no significant association with AST (*p* = 0.293).

## 4. Discussion

### 4.1. Summary of Principal Findings

Findings from this cross-sectional study suggest a possible association between occupational pesticide exposure and subtle alterations in serum biomarkers (AFP, total bilirubin, and indirect bilirubin). Importantly, because mean concentrations across all evaluated biomarkers remained within conventional clinical reference intervals in both groups, these differences likely represent mild and subclinical hepatic variations rather than overt hepatic dysfunction.

### 4.2. Comparison with Existing Literature

The transaminase patterns observed in this study broadly parallel international findings. Higher ALT and/or AST levels have been frequently documented among pesticide-exposed agricultural workers across diverse settings, including Thailand [10], Ethiopia [12], Cameroon [17,18], Tanzania [19], and India [20]. Conversely, other investigations, such as among Thai orchid growers [9] and agricultural workers in Saudi Arabia [21], identified less consistent hepatic responses. Such inter-study heterogeneity likely reflects variations in exposure intensity and duration, specific chemical formulations, protective equipment use, methodological designs, and potential host genetic factors in xenobiotic metabolism [22,23].

The divergence between a non-significant mean AST difference and a higher prevalence of individual abnormalities, alongside a duration-dependent gradient, may explain existing inconsistencies across studies. Transaminase shifts associated with pesticide exposure might not occur uniformly across an entire cohort; rather, they may emerge progressively, becoming noticeable at the aggregate level primarily among subgroups with longer exposure histories. This pattern aligns with findings from Thailand [11,24] and Ethiopia [12], where hepatic alterations were notably more pronounced in agricultural workers with prolonged exposure. Similarly, our observations of elevated bilirubin fractions parallel reports from greenhouse and occupational cohorts evaluating toxicological profiles [23,25]. Beyond Asia and Africa, comparable biomarker trends have been described in Ecuadorian rural communities [26] and synthesized in a Brazilian systematic review [27], pointing to a possible exposure–biomarker association across diverse agricultural settings.

### 4.3. Biological Plausibility

The subtle hepatic biomarker variations observed in this study are mechanistically plausible. As the primary site for xenobiotic biotransformation, the liver metabolizes diverse pesticide classes through the cytochrome P450 enzyme system. This process can generate reactive oxygen species and electrophilic intermediates that promote hepatocellular oxidative stress, mitochondrial impairment, and downstream cell death pathways [6,28]. Field studies have similarly documented elevated oxidative and genetic stress markers among workers handling organophosphates and pyrethroids [29]. Consequently, minor elevations in circulating cytosolic enzymes, notably ALT due to its higher liver specificity, likely reflect low-grade hepatocyte membrane permeability shifts rather than massive necrosis [8].

Although average AFP levels remained well below clinical diagnostic thresholds for hepatocellular carcinoma, the duration-dependent increase in AFP is noteworthy. Rather than indicating overt malignant transformation, this elevation may reflect low-grade, compensatory hepatocyte regeneration in response to repeated, low-dose toxicant exposure [6,7,30]. Furthermore, epidemiological links between chronic pesticide exposure and non-alcoholic fatty liver disease support a broader continuum of metabolic and cellular perturbations beyond isolated transaminase leakage [31,32]. In this context, the concurrent, duration-dependent rises in total and unconjugated bilirubin may suggest mild, subclinical inefficiencies in hepatocellular uptake or glucuronidation pathways, representing early functional alterations in hepatic handling. However, because sample collection coincided with the peak harvest season of local subtropical fruits (e.g., lychee), during which local fructose and polyphenol intake from fresh fruit consumption typically rises, seasonal dietary factors independently capable of modulating hepatic metabolism cannot be excluded as a contributor to the observed bilirubin and AFP elevations; these findings are therefore best interpreted as observed associations rather than direct, pesticide-specific causal effects.

### 4.4. Exposure–Response Association

Both pairwise comparisons and multivariable linear regression models showed that subtle biomarker variations were most apparent in farmers with >10 years of exposure. Nonetheless, given the cross-sectional design, convenience sampling, and potential residual confounding from seasonal factors during the fruit harvest, these patterns reflect non-causal statistical associations that warrant cautious interpretation.

### 4.5. Strengths of the Study

Key strengths of this study include the objective biochemical quantification of hepatic markers and the use of a geographically comparable reference group strictly free from smoking and alcohol confounders. Furthermore, our multi-step, label-verified exposure assessment effectively mitigated recall bias.

### 4.6. Limitations

Several methodological limitations should be acknowledged. First, the cross-sectional design precludes causal inference and cannot establish temporal sequence or rule out reverse causation. Second, occupational exposure was assessed via questionnaires rather than internal dose biomonitoring; consequently, findings reflect broad occupational associations rather than compound-specific toxicological responses [33]. Third, the convenience sampling strategy, modest sample size (n = 150), and unequal group allocation (50 unexposed controls vs. 100 exposed farmers) may introduce selection bias and limit statistical power for subtle subgroup comparisons. Fourth, despite screening out individuals with known liver disorders, chronic medications, and metabolic comorbidities, residual confounding from subclinical viral hepatitis (without testing for HBV/HCV serology), nutritional variations, or concurrent environmental co-exposures cannot be fully ruled out. Fifth, data collection occurred during the peak harvest season of local subtropical fruits (e.g., lychee). Substantial seasonal increases in dietary fructose and polyphenols from fresh fruit consumption could transiently alter hepatic metabolism and bilirubin kinetics, constituting a potential unmeasured nutritional confounder in this cross-sectional framework. Sixth, because farmers routinely handled complex, seasonal mixtures of active ingredients, this study cannot isolate the independent contributions of specific chemical classes or formulations. Finally, the external validity of these findings beyond this specific fruit-farming context warrants caution, given regional disparities in pesticide mixtures, spraying equipment, and protective practices, which can substantially modify biological exposure burdens across agricultural populations [34].

### 4.7. Public Health and Policy Implications

These findings suggest potential merit in periodic hepatic screening for long-tenured farmers (>10 years). In areas predominantly utilizing WHO Class II pesticides, preventive efforts could emphasize consistent personal protective equipment adherence and gradual adoption of Integrated Pest Management (IPM) alongside safer biopesticides [35], supported by targeted extension training and regulatory pathways [36]. Given that oxidative pathways are broadly implicated across pesticide classes [28,37], future public health initiatives would benefit from addressing overall cumulative chemical burdens rather than viewing hepatic parameters in isolation.

## 5. Conclusions

These results highlight potential duration-related subclinical liver biomarker shifts in occupationally exposed farmers. Structured health surveillance at the primary care level, coupled with accessible biochemical screening, could aid in early risk identification among long-term workers. Concurrently, public health efforts should prioritize actionable, competency-based interventions, such as supervised personal protective equipment (PPE) training, alongside broader chemical safety education.

## Figures and Tables

**Table 1:** Characteristics of the study population.

Characteristics		Exposed Group (n = 100)	Unexposed Group (n = 50)	*p*-Value
Age (years)	Mean ± SD	49.38 ± 10.19	48.90 ± 5.51	0.734
	Range	25.0–60.0	33.0–55.0	
BMI (kg/m^2^)	Mean ± SD	22.81 ± 2.78	22.68 ± 2.66	0.782
	Range	15.82–30.22	14.26–29.32	
BMI category	Normal (18.5–24.9)	80 (80.0%)	44 (88.0%)	0.221 †
	Underweight (<18.5)	8 (8.0%)	1 (2.0%)	
	Overweight (≥25)	12 (12.0%)	5 (10.0%)	
Sex	Male	20 (20.0%)	16 (32.0%)	0.096
	Female	80 (80.0%)	34 (68.0%)	
Smoking status	Non-smokers	100 (100.0%)	50 (100.0%)	—
Alcohol use	Non-drinkers	100 (100.0%)	50 (100.0%)	—
Marital status	Married	94 (94.0%)	45 (90.0%)	0.386
	Single	6 (6.0%)	5 (10.0%)	
Education	Primary school	31 (31.0%)	—	
	Secondary school	58 (58.0%)	—	
	High school	11 (11.0%)	—	
	College/University	—	50 (100.0%)	
Local residence	>10 years	100 (100.0%)	50 (100.0%)	—

Age and BMI *p*-values calculated using the independent-samples (Welch) *t*-test; sex and marital status p-values calculated using the chi-square test. † BMI category comparison calculated using the chi-square test across three categories. SD, standard deviation; BMI, body mass index.

**Table 2:** Characteristics of occupational exposure (n = 100).

Category/Variables	Specification	n (%)
Fruit farm area (hectare)	≤1	90 (90.0%)
	>1	10 (10.0%)
Pesticide use	Pesticide users	100 (100.0%)
Farming experience (years)	<5	35 (35.0%)
	5–10	40 (40.0%)
	>10	25 (25.0%)
Pesticide spraying frequency (times/season)	<5	9 (9.0%)
	5–10	45 (45.0%)
	>10	46 (46.0%)
Individual PPE Components (Always/Usually used)	Masks/Respirators	100 (100.0%)
	Protective gloves	92 (92.0%)
	Rubber boots	92 (92.0%)
	Protective clothing	47 (47.0%)
	Protective hats/Head coverings	96 (96.0%)
	Eye goggles/Face shields	11 (11.0%)
	Adequate Protective Practice (all 6 items used consistently)	10 (10.0%)

**Table 3:** Toxicological classification of pesticide products used in fruit farms, according to WHO guidelines.

Type	Brand Name	Active Ingredient(s)	WHO Hazard Classification
Insecticide	Actatoc 200WP	Acetamiprid	Class II
Insecticide	Repdor 250EC	Quinalphos, lambda-cyhalothrin	Class II
Insecticide	Than chop	Chlorfluazuron, thiamethoxam, profenofos	Class II
Insecticide	Shepatin 75EC	Abamectin, alpha-cypermethrin	Class II
Insecticide	Tanwin 4.0EC	Emamectin benzoate/avermectin	Biological
Insecticide	Fastphos 50EC	Alpha-cypermethrin	Class II
Insecticide	Scorpion 36EC	Abamectin, fipronil	Class II
Insecticide	Rocketasia 650EC	Chlorpyrifos ethyl, alpha-cypermethrin, fenobucarb	Class II
Insecticide	Emetin 1.9EC	Emamectin benzoate	Biological
Insecticide	Haihamec 1.8EC	Abamectin	Biological
Insecticide	Crymerin 100EC	Permethrin	Class II
Insecticide	Sapen Alpha 5EC	Alpha-cypermethrin	Class II
Insecticide	VK.Superlau 400SC	Buprofezin, imidacloprid	Class II
Insecticide	Map-Judo	Buprofezin	Class II
Insecticide	Tasieu 1.0EC	Avermectin B1a/B1b	Class II
Insecticide	Dratoc 666EC	Fenobucarb, chlorpyrifos ethyl, lambda-cyhalothrin	Class II
Insecticide	Lanomyl 680WP	Mancozeb, metalaxyl-M	Class II
Insecticide	Elixir 750WG	Chlorothalonil, mancozeb	Class II
Fungicide	Crystalusa 450SC	Azoxystrobin, metalaxyl	Class II
Fungicide	Majetic Top 420SC	Azoxystrobin, difenoconazole	Class II
Fungicide	Asmiltatop Super 400SC	Azoxystrobin, difenoconazole	Class II
Fungicide	Carbenzim 500FL	Carbendazim	Class II
Fungicide	Dosay 45WP	Cymoxanil, zineb, copper oxychloride	Class II
Herbicide	Co Chay	Glufosinate-ammonium	Class II
Herbicide	Khai Hoang Q7	Glufosinate-ammonium	Class II
Regulator	Poly-Liquid	Phospholipid, N, P, K, Mn, Cu, Zn, vitamin B6	Not classified
Regulator	A-K 205	N, P, K, Mg, Cu, B	Not classified

WHO hazard classification per the WHO Recommended Classification of Pesticides by Hazard [16]. “Biological” denotes avermectin/emamectin-benzoate-derived biopesticides, classified separately from conventional chemical hazard classes.

**Table 4:** Biomarker levels of exposed and unexposed groups.

Biomarkers	Parameters	Exposed Group (n = 100)	Unexposed Group (n = 50)	Reference Range	*p*-Value
	Mean ± SD	5.11 ± 0.91	5.29 ± 0.66	3.9–6.4	0.170
Glucose (mmol/L)	Range	3.92–10.14	4.41–8.56		
	Abnormal, n (%)	5 (5.0%)	2 (4.0%)		0.792
	Mean ± SD	34.54 ± 10.66	30.06 ± 13.37	<37	0.105
AST (U/L)	Range	17.10–99.10	17.40–88.40		
	Abnormal, n (%)	23 (23.0%)	4 (8.0%)		0.043 *
	Mean ± SD	28.88 ± 11.06	22.36 ± 12.21	<40	0.002 **
ALT (U/L)	Range	17.10–118.80	10.50–99.10		
	Abnormal, n (%)	20 (20.0%)	3 (6.0%)		0.045 *
	Mean ± SD	3.06 ± 3.30	1.16 ± 1.09	<9	<0.001 ***
AFP (ng/mL)	Range	0.50–28.98	0.50–5.07		
	Abnormal, n (%)	7 (7.0%)	0 (0%)		0.096 (Fisher’s exact)
	Mean ± SD	9.93 ± 3.17	7.74 ± 3.06	<17	<0.001 ***
Total bilirubin (μmol/L)	Range	4.69–20.74	1.66–16.62		
	Abnormal, n (%)	5 (5.0%)	0 (0%)		0.170 (Fisher’s exact)
	Mean ± SD	8.40 ± 3.15	5.26 ± 2.75	<12.7	<0.001 ***
Indirect bilirubin (μmol/L)	Range	3.15–20.50	0.52–12.82		
	Abnormal, n (%)	6 (6.0%)	0 (0%)		0.179 (Fisher’s exact)

OR for abnormal ALT (exposed vs. unexposed) = 3.92 (95% CI 1.11–13.90), calculated by the Woolf method. OR undefined (infinite) owing to the zero-count cell in the non-exposed group; Haldane–Anscombe continuity-corrected OR ≈ 8.10, 5.82, 6.95 for AFP, Total bilirubin, Indirect bilirubin, respectively. * *p* < 0.05, ** *p* < 0.01, *** *p* < 0.001.

**Table 5:** Effect of pesticide exposure duration on liver biomarker levels among exposed farmers.

Biomarker	<5 Years	5–10 Years	>10 Years	ANOVA F (df = 2.97)	*p*-Value
AST (U/L)	31.06 ± 11.31	32.08 ± 12.32	36.74 ± 11.67	1.84	0.164
ALT (U/L)	25.28 ± 13.33	27.88 ± 12.26	31.98 ± 12.68	2.02	0.139
AFP (ng/mL)	1.06 ± 3.35	2.06 ± 4.20	7.08 ± 4.31	18.66	<0.001 ***
Total bilirubin (μmol/L)	8.91 ± 2.17	9.03 ± 3.11	10.92 ± 3.17	4.44	0.014 *
Indirect bilirubin (μmol/L)	6.40 ± 2.15	7.80 ± 4.23	8.90 ± 4.15	3.60	0.031 *

F-statistics and *p*-values calculated by one-way ANOVA (between-groups df = 2, within-groups df = 97). * *p* < 0.05, *** *p* < 0.001.

**Table 6:** Serum biomarker levels stratified by cumulative pesticide exposure duration.

Biomarker	<5 Years vs. 5–10 Years	<5 Years vs. >10 Years	5–10 Years vs. >10 Years
AFP	q = 1.55, ns	q = 8.23, p < 0.001	q = 7.05, p < 0.001
Total bilirubin	q = 0.26, ns	q = 3.83, p < 0.05	q = 3.70, p < 0.05
Indirect bilirubin	q = 2.37, ns	q = 3.73, p < 0.05	q = 1.69, ns

ns, not significant (q < q-critical = 3.37).

**Table 7:** Adjusted linear regression models of serum biomarker levels across pesticide exposure categories.

	Difference in Biomarker Levels ([95%CI], FDR-Adjusted *p*-Value)				
Exposure	ALT (U/L)	AST (U/L)	AFP (μg/L)	Total Bilirubin (mg/dL)	Indirect Bilirubin (mg/dL)
NE *	Ref	Ref	Ref	Ref	Ref
<5 years	1.20 ([−2.10, 4.50], 0.652)	0.90 ([−3.10, 4.90], 0.802)	0.05 ([−0.30, 0.40], 0.832)	0.02 ([−0.08, 0.12], 0.802)	−0.01 ([−0.10, 0.08], 0.832)
5–10 years	3.40 ([−0.80, 7.60], 0.293)	2.10 ([−2.50, 6.70], 0.617)	0.21 ([−0.15, 0.57], 0.473)	0.09 ([−0.04, 0.22], 0.379)	0.04 ([−0.07, 0.15], 0.652)
> 10 years	7.50 ([−0.90, 14.10], 0.060)	4.80 ([−1.20, 10.80], 0.293)	1.95 ([0.60, 3.30], **0.022**)	0.42 ([0.15, 0.69], **0.022**)	0.12 ([0.01, 0.23], **0.025**)

Abbreviations: FDR: false discovery rate, CI: confidence interval. Adjustments: Models are adjusted simultaneously for age, sex, BMI, smoking status, alcohol drinking status and other available covariates. * NE: Non-exposed group. Note: Values inside parentheses represent the unstandardized coefficient, followed by the bracketed 95% confidence interval and the Benjamini–Hochberg FDR- adjusted *p*-value (q-value). Bolded text highlights associations remaining statistically significant at FDR- adjusted *p* < 0.05.

## Data Availability

The data presented in this study are available on request from the corresponding author.

## Abbreviations

The following abbreviations are used in this manuscript: AFP, alpha-fetoprotein; ALT, alanine aminotransferase; AST, aspartate aminotransferase; BMI, body mass index; CI, confidence interval; CYP, cytochrome P450; IQC, internal quality control; OR, odds ratio; PPE, personal protective equipment; SD, standard deviation; SOP, standard operating procedure; STROBE, Strengthening the Reporting of Observational Studies in Epidemiology; WHO, World Health Organization.

## References

[1] Food and Agriculture Organization of the United Nations (2023). Pesticides Use and Trade 1990–2023.

[2] Phong L.T., Thong A.C. (2020). Highly Hazardous Pesticides in Vietnam: A Situational Analysis.

[3] Giang N.D., Nguyen V.H., Hoang T.L., Tran T.V.T., Huynh T.P.L., Nguyen T.Q.T. (2022). Assessment of pesticide use and pesticide residues in vegetables from two provinces in Central Vietnam. PLoS ONE.

[4] Nguyen T.M., Thanh N.T., Havukainen J., Hannaway D.B. (2018). Pesticide use in vegetable production: A survey of Vietnamese farmers’ knowledge. Plant Prot. Sci..

[5] Rizzati V., Briand O., Guillou H., Gamet-Payrastre L. (2016). Effects of pesticide mixtures in human and animal models: An update of the recent literature. Chem. Biol. Interact..

[6] Khairullina Z., Kurmangaliyeva S., Yussupov R., Kelimberdiyeva E., Tryfonyuk L., Shapambayev N., Seidakhmetova A., Medetbekov T., Tkachenko A. (2026). Pesticides drive liver diseases through non-apoptotic regulated cell death pathways. Diseases.

[7] VoPham T., Bertrand K.A., Hart J.E., Laden F., Brooks M.M., Yuan J.-M., Talbott E.O., Ruddell D., Chang C.-C.H., Weissfeld J.L. (2017). Pesticide exposure and liver cancer: A review. Cancer Causes Control.

[8] Li W., Xiao H., Wu H., Xu X., Zhang Y. (2022). Organophosphate pesticide exposure and biomarkers of liver injury/liver function. Liver Int..

[9] Aroonvilairat S., Kespichayawattana W., Sornprachum T., Chaisuriya P., Siwadune T., Ratanabanangkoon K. (2015). Effect of pesticide exposure on immunological, hematological and biochemical parameters in Thai orchid farmers—A cross-sectional study. Int. J. Environ. Res. Public Health.

[10] Bunsri S., Muenchamnan N., Naksen W., Ong-Artborirak P. (2023). The hematological and biochemical effects from pesticide exposure on Thai vegetable farmers. Toxics.

[11] Kopolrat K.Y., Chaiklieng S., Sithithaworn P., Suggaravetsiri P., Pruktharathikul V., Trinnawoottipong K. (2025). Associations between pesticide exposure and hepatobiliary disease among agricultural workers in the Northeastern region of Thailand: A cross-sectional study. J. Occup. Med. Toxicol..

[12] Gietie H.M., Dedefo G., Alem M., Wolde G., Aliye M., Disani A., Habtu W., Ashebir G., Minlarg E., Bitew T. (2026). Effect of pesticide exposure on liver function tests and serum cholinesterase levels among floriculture industry workers in Bahir Dar, Ethiopia: A comparative cross-sectional study. Sci. Rep..

[13] Ngoan L.T., Lua N.T., Hang L.T.M. (2007). Cancer mortality pattern in Viet Nam. Asian Pac. J. Cancer Prev..

[14] Dang H.V., Nguyen L.T., Tran H.T., Nguyen H.T., Dang A.K., Ly V.D., Frazzoli C. (2017). Risk Factors for Non-communicable Diseases in Vietnam: A Focus on Pesticides. Front. Environ. Sci..

[15] Beier J.I., Luo J., Vanderpuye C.M., Brizendine P., Muddasani P., Bolatimi O., Heinig S.A., Ekuban F.A., Siddiqui H., Ekuban A. (2025). Environmental Pollutants, Occupational Exposures, and Liver Disease. Semin. Liver Dis..

[16] World Health Organization (2020). The WHO Recommended Classification of Pesticides by Hazard and Guidelines to Classification, 2019.

[17] Manfo F.P.T., Mboe S.A., Nantia E.A., Ngoula F., Telefo P.B., Moundipa P.F., Cho-Ngwa F. (2020). Evaluation of the effects of agro pesticides use on liver and kidney function in farmers from Buea, Cameroon. J. Toxicol..

[18] Nanhah V.J.K., Ntepe L.J.M., Tchuente B.R.T., Hagbe P.V., Goda D., Ekedjoum Y.D.E., Ella F.A., Nguemto G.R.T., Mandob D.E., Ngondi J.L. (2025). Neurological and organ health risks associated with pesticide mixture exposure in banana farm workers in Moungo Division, Cameroon. Sci. Rep..

[19] Manyilizu W.B., Mdegela R.H., Kazwala R., Nonga H., Muller M., Lie E., Skjerve E., Lyche J.L. (2016). Association of long-term pesticide exposure and biologic parameters in female farm workers in Tanzania: A cross-sectional study. Toxics.

[20] Patil J.A., Patil A.J., Sonttake A., Govindwar S.P. (2009). Occupational pesticides exposure of sprayers of grape gardens in western Maharashtra (India): Effects on liver and kidney function. J. Basic Clin. Physiol. Pharmacol..

[21] Al-Sarar A.S., Bakr Y.A., Hussein H.I., Bayoumi A.E. (2009). Hematological and biochemical alterations in occupationally pesticide-exposed workers of Riyadh municipality, Kingdom of Saudi Arabia. Res. J. Environ. Toxicol..

[22] Hernández A.F., Gil F., Lacasaña M., Rodríguez-Barranco M., Tsatsakis A.M., Requena M., Parrón T., Alarcón R. (2013). Pesticide exposure and genetic variation in xenobiotic-metabolizing enzymes interact to induce biochemical liver damage. Food Chem. Toxicol..

[23] García-García C.R., Parrón T., Requena M., Alarcón R., Tsatsakis A.M., Hernández A.F. (2016). Occupational pesticide exposure and adverse health effects at the clinical, hematological and biochemical level. Life Sci..

[24] Sombatsawat E., Barr D.B., Panuwet P., Robson M.G., Siriwong W. (2021). Pesticide-induced changes in cholinesterase activity and chronic kidney disease of unknown etiology among farmers in Nakhon Ratchasima, Thailand. Hum. Ecol. Risk Assess..

[25] Lozano-Paniagua D., Parrón T., Alarcón R., Requena M., López-Guarnido O., Lacasaña M., Hernández A.F. (2021). Evaluation of conventional and non-conventional biomarkers of liver toxicity in greenhouse workers occupationally exposed to pesticides. Food Chem. Toxicol..

[26] Mehta P., Parajuli R.P., Chronister B.N.C., Yang K., Barr D.B., Tu X.M., Lopez-Paredes D., Suarez-Lopez J.R. (2025). Pesticide and liver biomarkers among Ecuadorian adolescents and adults living in agricultural settings. Toxics.

[27] Panis C., Kawassaki A.C.B., Crestani A.P.J., Pascotto C.R., Bortoloti D.S., Vicentini G.E., Lucio L.C., Ferreira M.O., Prates R.T.C., Vieira V.K. (2022). Evidence on human exposure to pesticides and the occurrence of health hazards in the Brazilian population: A systematic review. Front. Public Health.

[28] Sule R.O., Condon L., Gomes A.V. (2022). A common feature of pesticides: Oxidative stress—The role of oxidative stress in pesticide-induced toxicity. Oxid. Med. Cell. Longev..

[29] Zepeda-Arce R., Rojas-García A.E., Benitez-Trinidad A., Herrera-Moreno J.F., Medina-Díaz I.M., Barrón-Vivanco B.S., Villegas G.P., Hernández-Ochoa I., Heredia M.d.J.S., Bernal-Hernández Y.Y. (2017). Oxidative stress and genetic damage among workers exposed primarily to organophosphate and pyrethroid pesticides. Environ. Toxicol..

[30] Ezzat S., Abdel-Hamid M., Eissa S.A., Mokhtar N., Labib N.A., El-Ghorory L., Mikhail N.N., Abdel-Hamid A., Hifnawy T., Strickland G.T. (2005). Associations of pesticides, HCV, HBV, and hepatocellular carcinoma in Egypt. Int. J. Hyg. Environ. Health.

[31] Sang H., Lee K.-N., Jung C.H., Han K., Koh E.H. (2022). Association between organochlorine pesticides and nonalcoholic fatty liver disease in the National Health and Nutrition Examination Survey 2003–2004. Sci. Rep..

[32] Yang J.S., Park Y. (2018). Insecticide exposure and development of nonalcoholic fatty liver disease. J. Agric. Food Chem..

[33] Martin-Reina J., Casanova A.G., Dahiri B., Fernández I., Fernández-Palacín A., Bautista J., Morales A.I., Moreno I. (2021). Adverse health effects in women farmers indirectly exposed to pesticides. Int. J. Environ. Res. Public Health.

[34] Negatu B., Kromhout H., Mekonnen Y., Vermeulen R. (2016). Use of chemical pesticides in Ethiopia: A cross-sectional comparative study on knowledge, attitude and practice of farmers and farm workers in three farming systems. Ann. Occup. Hyg..

[35] Reddy A.A., Reddy M., Mathur V. (2024). Pesticide use, regulation, and policies in Indian agriculture. Sustainability.

[36] Reddy A.A., Mouzam S.M., Praveen K.V., Mohan G. (2026). Biopesticide market and regulatory landscape with determinants of farm-level use in India. Discov. Sustain..

[37] Damalas C.A., Koutroubas S.D. (2016). Farmers’ exposure to pesticides: Toxicity types and ways of prevention. Toxics.

